# A covalent inhibitor targeting Cys-349 of LIMK1 confers selectivity over LIMK2

**DOI:** 10.1016/j.jbc.2026.113322

**Published:** 2026-07-13

**Authors:** Jon B. Patteson, Ioannis Manolaridis, Mee Ra Hong, Jennifer M. Johnston, Samaneh Mesbahi-Vasey, Michael C. Gregory, Daniel V. Iwamoto, John C. Reid, John M. Sanders, Ditte Lovatt, Terrence P. McDonald, Valerie W. Shurtleff, Sandra B. Gabelli, Marina Bukhtiyarova

**Affiliations:** 1 Quantitative Biosciences, Merck & Co, Inc, MRL, West Point, Pennsylvania, USA; 2 Discovery Chemistry, Merck & Co, Inc, MRL, West Point, Pennsylvania, USA; 3 Neuroscience, Merck & Co, Inc, MRL, West Point, Pennsylvania, USA

**Keywords:** crystallography, inhibition mechanism, kinase, pharmacology, SPR

## Abstract

LIM domain kinase 1 (LIMK1) has been identified as a promising therapeutic target for a variety of conditions, such as chronic pain, open-angle glaucoma, various cancers, schizophrenia, and Fragile X syndrome. However, identifying inhibitors that selectively inhibit LIMK1 over LIM domain kinase 2 (LIMK2) has proven to be challenging. A viable strategy to overcome this difficulty is the development of covalent inhibitors, which can offer both potency and selectivity for LIMK1 due to a reactive cysteine, C349, near the active site absent in its paralog LIMK2. Here we identify an irreversible covalent inhibitor of LIMK1 (cLIMK1i), which is highly selective for LIMK1 over both LIMK2 and a panel of over 100 kinases. A crystal structure of LIMK1 soaked with cLIMK1i reveals it is a type I inhibitor occupying the ATP-binding site with its acrylamide moiety oriented toward the P-loop where C349 resides. Computational modeling supports that the P-loop of LIMK1 can adopt a conformation compatible with covalent bond formation. Biochemical and biophysical characterization of the interaction of cLIMK1i with LIMK1 demonstrates that the covalent bond with LIMK1-C349 is essential for its potent inhibition. These results support covalent inhibition of LIMK1 as a viable strategy for selectively inhibiting LIMK1 over LIMK2 and other kinases.

LIM domain kinases 1 and 2 (LIMK1 and LIMK2) are closely related serine/threonine kinases that regulate actin dynamics by phosphorylating and inactivating cofilin isoforms (1, 2). LIMK1 and LIMK2 are activated by multiple upstream kinases at several characterized sites (3, 4). Once activated, LIMK1 and LIMK2 phosphorylate and inactivate their substrates cofilin-1 and cofilin-2, which decreases actin dynamics (1, 5). Decreased actin dynamics directly affect cell motility, proliferation, differentiation and apoptosis as well as synapse stability, and neuritogenesis (2, 4, 6). The multitude of downstream effects of LIMK1 and LIMK2 activity have implicated these kinases in many pathologies including cancers (7), viral infection (8), glaucoma (9), pain (10), and neuronal diseases (11, 12).

Identification of selective LIMK1 and LIMK2 inhibitors can be useful for specifically modulating the functions of each kinase paralog. LIMK1 and LIMK2 share 50% sequence identity overall, 70% in the kinase domain (KD), and 90% in the active site (Fig. S1) (6). The high similarity between LIMK1 and LIMK2 has, therefore, made the discovery of selective active site binders challenging (13). One strategy for identifying a selective LIMK1 inhibitor is to target cysteine-349 (C349), which is located on the P-loop of LIMK1 positioned near the ATP-binding site. Critically, no cysteine is present on this loop in LIMK2.

Selective inhibition of kinases by exploiting reactive cysteines has shown promise for several other kinase drug targets, including several clinically used covalent kinase inhibitors such as those targeting BTK (Ibrutinib) and EGFR (Osimertinib) (14, 15, 16). For applying this covalent inhibition strategy to LIMK1, very recently a covalent LIMK1 inhibitor SM311 was identified by adding a covalent warhead to a compound, which, without the warhead, inhibited LIMK1 and LIMK2 indiscriminately (17). To further support this covalent inhibition strategy of LIMK1, a covalent kinase probe molecule SMI-71 has been shown to bind LIMK1 in tissue samples; however, this compound demonstrated poor kinome selectivity, binding to at least 16 other kinases (18).

Here we report the discovery of a highly selective covalent LIMK1 inhibitor (cLIMK1i). cLIMK1i shows strong selectivity for LIMK1 over LIMK2 and excellent kinome-wide selectivity. Biophysical, structural, and computational analyses demonstrate that cLIMK1i irreversibly covalently modifies C349, and that this covalent engagement of C349 is the principal determinant of its selectivity for LIMK1.

## Results

### Identification of cLIMK1i

**Figure 1:**
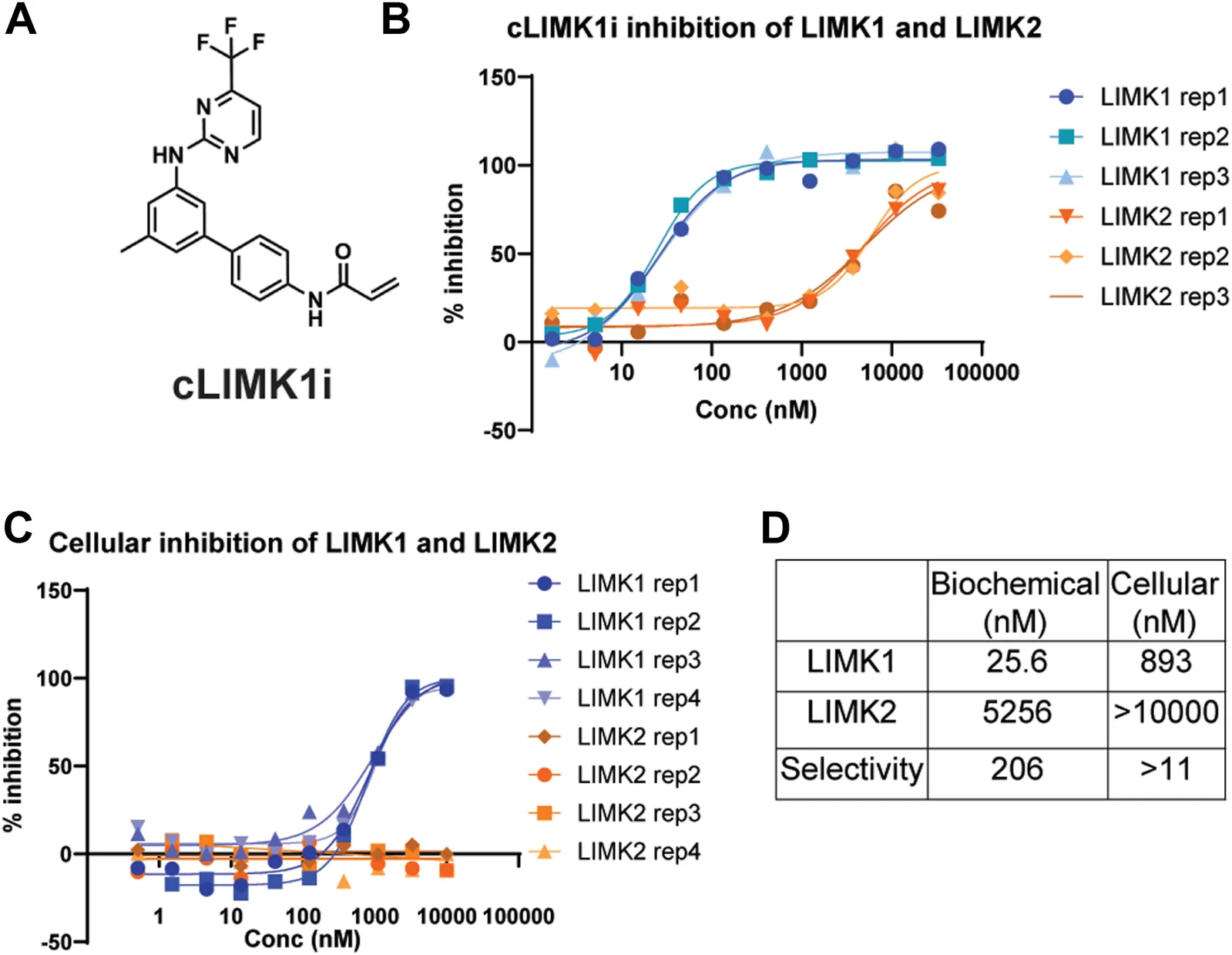
**Identification of cLIMK1i as a selective LIMK1 inhibitor over LIMK2.***A*, structure of cLIMK1i. *B*, dose response curves for cLIMK1i inhibition of LIMK1 and LIMK2 in enzymatic format. Assay measures the inhibition of the phosphorylation of the substrate cofilin-1. *C*, dose response curves for cLIMK1i inhibition of LIMK1 and LIMK2 over-expressed in HEK293T cells. Assay measures the inhibition of the phosphorylation of the substrate cofilin-1. *D*, comparison of IC_50_ values (in nM) for cLIMK1i inhibition of LIMK1 and LIMK2, and selectivity ratios. cLIMK1i, covalent inhibitor of LIMK1; IC_50_, half maximal inhibitor concentration; nM, nanomolar; LIMK1, LIM domain kinase 1; LIMK2, LIM domain kinase 2.

To identify LIMK1 inhibitors, we assessed internal chemical matter from previous kinase programs that had been counter-screened against a panel of 124 kinases which included LIMK1, selecting for compounds which displayed 50% inhibition or 50% binding to LIMK1 at 1 μm. These potential LIMK1 inhibitors were then re-assessed for inhibition of phosphorylation of cofilin-1 by LIMK1 and LIMK2 in cascade assay format, which uses an upstream kinase to activate LIMK1/2 *in situ* and enables the identification of compounds that inhibit LIMK1/2 in the activated and/or inactivated states, a strategy which has been used successfully to identify unique inhibitors for other kinases (Fig. S2) (19, 20, 21). One compound, cLIMK1i, stood out with an half maximal inhibitor concentration (IC_50)_ of 26 ± 1 nanomolar (nM)against LIMK1 and an IC_50_ of 5300 ± 500 nM against LIMK2—206-fold selectivity for inhibition of LIMK1 over LIMK2 (Fig. 1, *A* and *B*). To our knowledge, this is the most selective inhibitor for LIMK1 over LIMK2 to date, showing substantially more selectivity over noncovalent LIMK1 inhibitors, and slightly more selectivity over the recently published covalent LIMK1 inhibitor (17, 22). When tested against a panel of 124 kinases at 1 μm, cLIMK1i showed ≥50% effect against just three other kinases, IRAK1, SYK, and CDK7 (Fig. S3). cLIMK1i also shows high selectivity for inhibition of LIMK1 over LIMK2 in a HEK293T cellular assay when overexpressing either LIMK1 or LIMK2 and measuring downstream cofilin-1 phosphorylation. cLIMK1i demonstrated an IC_50_ at 893 ± 105 nM in LIMK1-overexpressing cells in contrast to no measurable inhibition of LIMK2-overexpressing cells up to 10 μm as the top concentration tested. This reflects greater than 11-fold selectivity for LIMK1 over LIMK2 inhibition in the cellular setting (Fig. 1, *C* and *D*).

### cLIMK1i is an irreversible covalent LIMK1 inhibitor which binds LIMK1-C349

**Figure 2:**
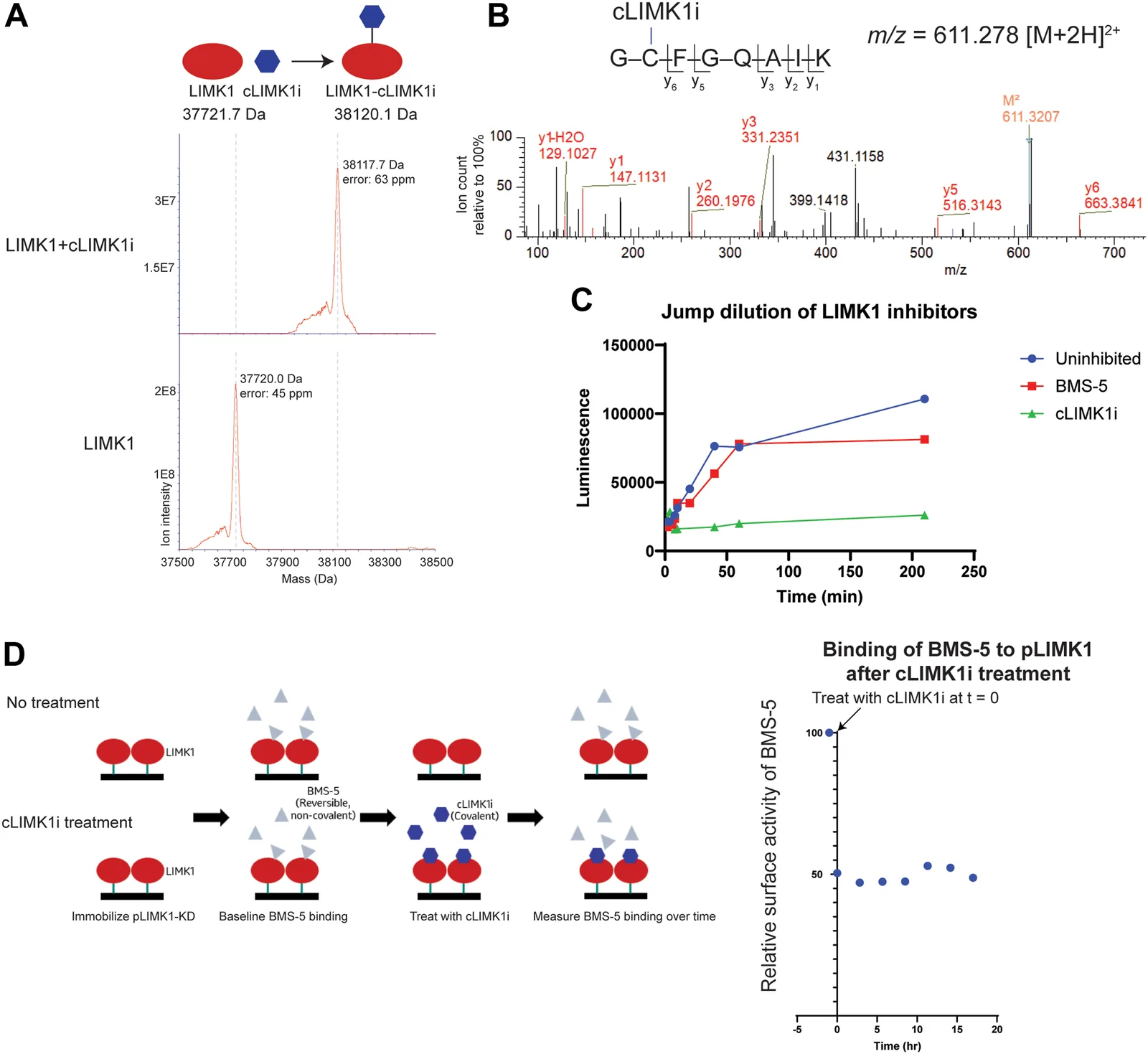
**cLIMK1i is an irreversible covalent inhibitor of LIMK1.***A*, deconvoluted mass spectra of intact mass data from LIMK1-KD treated with cLIMK1i. Mass shift of LIMK1 occurs in presence of cLIMK1i. *B*, LC-MS/MS detection of cLIMK1i bound to the peptide fragment bearing C349. Fragments which could be identified are highlighted and described by their fragmentation pattern. *C*, jump dilution of cLIMK1i inhibition. Jump dilution of cLIMK1i inhibition of pLIMK1 shows limited reversibility of cLIMK1i inhibition of pLIMK1-KD at up to 3 h after treatment. cLIMK1i was incubated with pLIMK1 at 75x above the IC_50_ determined from the cascade assay, and BMS-5 (known noncovalent inhibitor) was incubated with pLIMK1 at 16 μM (457× IC_50_ from the cascade assay). Samples were diluted to 1 nM pLIMK1 at assay start upon addition of ATP and cofilin-1. Final concentrations of cLIMK1i and BMS-5 were 4 nM (0.15× IC_50_) and 33 nM (0.94× IC_50_). *D*, cLIMK1i reversibility experiment measured with SPR. cLIMK1i is covalently bound to pLIMK1-KD, and BMS-5, a type I inhibitor, is flowed over the surface of pLIMK1-KD either treated or not treated with cLIMK1i. Relative surface activity is the maximum response of BMS-5 binding to cLIMK1i-treated surface divided by the maximum response of BMS-5 binding to untreated surface at each timepoint. BMS-5 unable to bind pLIMK1-KD up to 17 h after pretreatment with cLIMK1i. Additional information can be found in Figure S6. cLIMK1i, covalent inhibitor of LIMK1; IC_50_, half maximal inhibitor concentration; nM, nanomolar; SPR, surface plasmon resonance.

The presence of an acrylamide on cLIMK1i, in addition to the high selectivity for LIMK1 over LIMK2 inhibition, strongly suggested that cLIMK1i inhibits LIMK1 *via* a covalent mechanism. We assessed covalent bond formation of cLIMK1i to LIMK1-KD by intact MS. Incubation of cLIMK1i and LIMK1-KD, followed by intact MS showed an addition of 398 Da to the mass of LIMK1-KD, which corresponds to the addition of one cLIMK1i molecule (Fig. 2*A*). Similar reaction kinetics were observed with LIMK1-KD and pLIMK1-KD (phosphorylated on Threonine 508), as determined by intact MS, supporting no preference of cLIMK1i to inhibit phosphorylated or unphosphorylated LIMK1-KD (Fig. S4). To identify the site of covalent modification by the compound, cLIMK1i-treated LIMK1-KD was subjected to tryptic digestion, followed by LC-MS/MS analysis and peptide sequence analysis. A single peptide fragment, which includes C349, was identified as the only peptide with an additional mass corresponding to cLIMK1i (Fig. 2*B*). Fragmentation data strongly supports the identity of this peptide with modification at C349, such as the y^6^+ fragment which would require cLIMK1i to bind either to G348 or C349. As a control, the cLIMK1i adduct on this peptide was not identified when digesting LIMK1-KD without cLIMK1i treatment. Therefore, we confirmed the identity of cLIMK1i as a covalent inhibitor and identified the peptide on LIMK1 with which cLIMK1i reacts to form a stable covalent adduct.

Following confirmation of covalent binding of cLIMK1i to LIMK1, we investigated the inhibition kinetics of cLIMK1i. A reaction time course of pLIMK1-KD phosphorylation of cofilin-1 was measured using varying concentrations of cLIMK1i to assess whether the reaction kinetics support a reversible or irreversible inhibition mechanism (Fig. S5). The reaction progress stops entirely with as low as 250 nM cLIMK1i, supporting an irreversible covalent inhibition mechanism. A jump dilution experiment was also conducted to assess reversibility. At up to 3.5 h, minimal reversibility was measured (Fig. 2*C*).

To assess a longer time scale that might provide a more conclusive measure of reversibility, a surface plasmon resonance (SPR)-based experiment was conducted (23). Briefly, biotinylated-pLIMK1-KD was immobilized on a neutravidin surface, and a known LIMK1 inhibitor, BMS-5 (24), which is competitive with respect to cLIMK1i, was flowed over the surface to establish the baseline binding response to pLIMK1-KD, which we called 100% BMS-5 binding. Subsequently, either cLIMK1i or buffer was flowed over the surface of pLIMK1-KD for 15 min to enable either formation of a covalent bond with pLIMK1-KD, or leaving the surface uninhibited. Over the next 17 h, BMS-5 was intermittently flowed over the surface of pLIMK1-KD bound to cLIMK1i (or pLIMK1-KD treated with buffer) at intervals of 170 min for 90 s to measure any wash out of cLIMK1i from pLIMK1-KD. A recovery of BMS-5 binding levels on the cLIMK1-treated surface would indicate that binding of cLIMK1i is reversible; however, the results unambiguously show that after cLIMK1i binds pLIMK1-KD, no recovery of BMS-5 binding was observed for up to 17 h (Figs. 2*D*, S6). Therefore, the SPR-based experiment supports the findings from the biochemical assays that cLIMK1i forms an irreversible covalent bond with LIMK1.

### Crystal structure of LIMK1-KD^D460N^ bound to cLIMK1i

**Figure 3:**
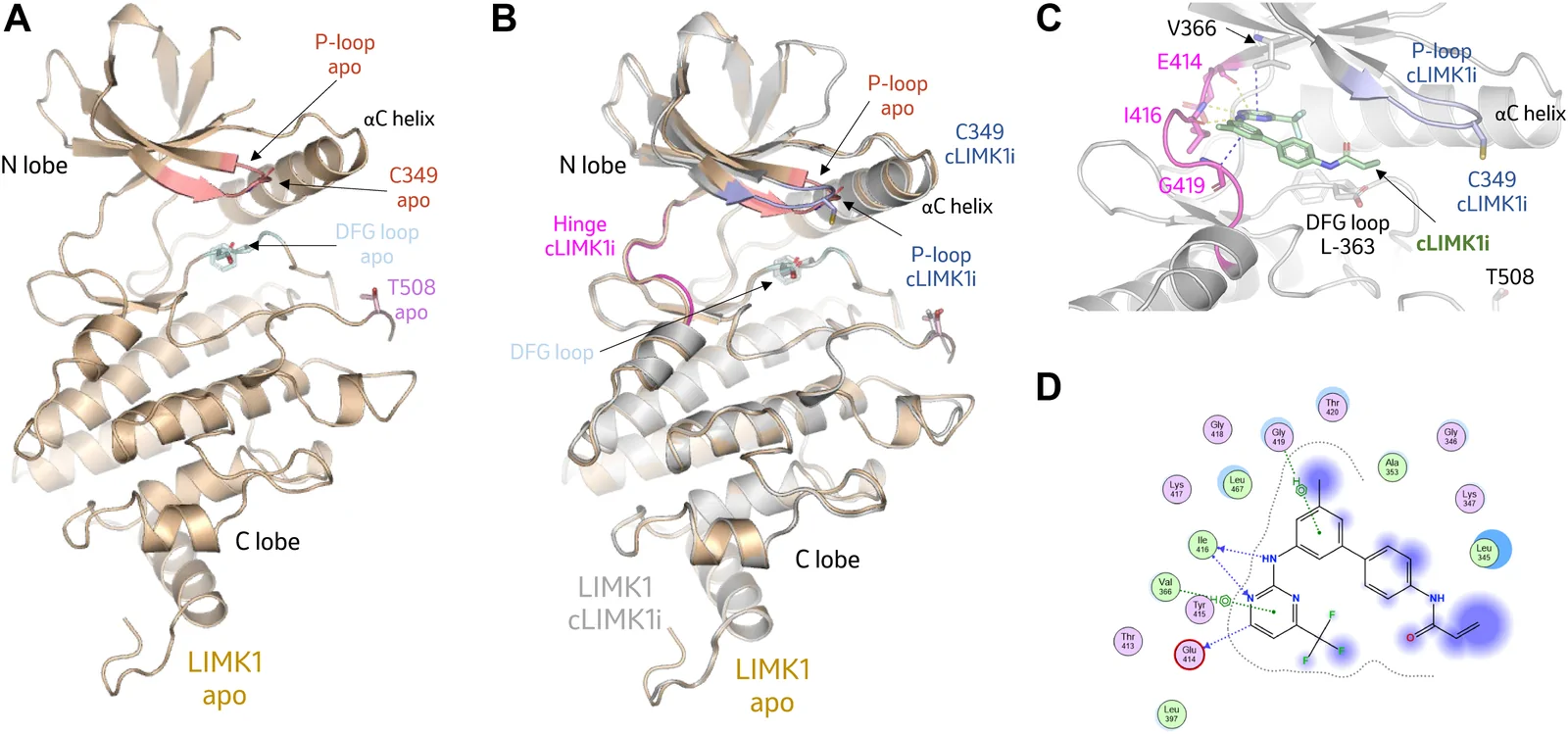
**Crystal structures of LIMK1-KD^D460N^ in both apo form and when bound to cLIMK1i.***A*, the apo LIMK1-KD^D460N^ adopts a canonical active-kinase conformation, characterized by a conformationally flexible P-loop (including C349). The DFG loop is in the 'in' position, and clear electron density is observed for T508, which remains unphosphorylated. *B*, an overlay of the apo LIMK1-KD^D460N^ crystal structure (PDB 9Z6E) with the cLIMK1i-bound complex (PDB 9Z6D) highlights the major regulatory elements within the LIMK1 catalytic core. For clarity, cLIMK1i has been removed from this overlay. Notably, the N- and C-lobes of both structures align very well, with no conformational changes observed due to inhibitor binding. *C*, the LIMK1-cLIMK1i complex exhibits a canonical active-kinase conformation, with the inhibitor positioned at the ATP-binding site, interacting with the LIMK1 hinge region through hydrogen bonding, classifying it as a type I inhibitor. The zoomed-in view of the binding site reveals the hydrogen bonds depicted as *blue dotted lines*, along with the position of C349 in the P-loop. *D*, an interaction diagram illustrates the binding mode of cLIMK1i to the type I site of LIMK1. cLIMK1i, covalent inhibitor of LIMK1; LIMK1, LIM domain kinase 1.

A crystal structure of cLIMK1i bound to LIMK1 would offer valuable insights into the binding mode of compounds located near, and potentially interacting with, C349. To facilitate this analysis, we developed a novel crystallization system for LIMK1 that reliably produces high-quality apo LIMK1-KD D460N (LIMK1-KD^D460N^) crystals. Even though the D460N mutation has been reported to abolish catalytic activity (25), we observed higher expression levels and superior stability of LIMK1-KD^D460N^ than WT. This effort resulted in the successful determination of the first reported high-resolution crystal structure of apo LIMK1 (Fig. 3*A*, PDB: 9Z6E, and Table 1), although there are reported structures of LIMK1 bound to type I, type II, and type III inhibitors (26, 27, 28). LIMK1-KD^D460N^ adopts a classic active-like conformation, distinguished by an ordered DFG motif situated within the ATP-binding pocket (DFG-in). The activation loop remains relatively extended, allowing space for ATP to bind, and the αC helix adopts an ‘in’ position, facilitating proper alignment of key catalytic residues and priming LIMK1 for activation upon ligand or substrate binding. In the apo LIMK1-KD^D460N^ crystal structure, the P-loop (including C349) is not fully visible (specifically residues C349-G351) at 2*mFo*-*DFc* contoured at 1.0 sigma and therefore has been partially modeled (C_α_), adopting an extended conformation (Fig. 3*A*).

**Table 1:** Data collection and refinement statistics. ∗Parentheses indicate higher resolution shell; statistics for both datasets derived from the ‘STARANISO’ output

Information	LIMK1-KD^D460N^ (apoapo)	LIMK1-KD^D460N^ + cLIMK1i
PDB accession code	9Z6E	9Z6D
Data collection		
Space group	*P*2_1_	*P*2_1_
X-ray source	CLS, CMCF-BM	CLS, CMCF-BM
Number crystals	1	1
Cell dimensions, *a*, *b*, *c* (Å)	54.3, 59.4, 103.0	54.2, 59.5, 101.6
*α, β, γ* (°)	90.0, 102.6, 90.0	90.0, 102.0, 90.0
Wavelength	1.18079	1.18061
Resolution range (Å) ∗	100.52–2.26 (2.46–2.26)	51.45–1.94 (2.15–1.94)
No. unique reflections	20,198	32,077
Degree of data (°)	220	360
Completeness (spherical) (%) ∗	66.7 (14.8)	67.6 (12.5)
Completeness (ellipsoidal) (%) ∗	90.4 (46.3)	89.8 (43.9)
*R*_*merge*_ ∗	0.081 (0.687)	0.045 (0.685)
*R*_*pim*_ (%) ∗	0.046 (0.486)	0.019 (0.396)
*I/σI* ∗	12.6 (1.4)	18.5 (1.5)
Multiplicity ∗	3.9 (2.6)	6.3 (3.8)
CC half ∗	0.99 (0.56)	0.99 (0.71)
Refinement		
Resolution range (Å) ∗	51.84–2.26 (2.38–2.26)	51.07–1.94 (2.00–1.94)
*R*_work_/*R*_free_ (%)	0.1918/0.2452	0.1874/0.2239
Total number of reflections	20,198	32,077
Test set number of reflections	1015	1592
Number of atoms _(macromolecule)_	4392	4365
Number of atoms _(ligand)_	-	58
Number of atoms _(waters)_	86	181
Model Quality		
RMSD bond lengths (Å)	0.0098	0.008
RMSD bond angles (°)	1.45	1.076
Average B-factor _(macromolecule)_	49.2	47.7
Average B-factor _(ligands)_	-	54.6
Average B-factor _(waters)_	42.6	45.4
Clash score	10.28	12.08
Ramachandran outliers (%)	1.11	0.37
Ramachandran allowed (%)	4.24	3.67
Ramachandran favored	94.65	95.96
MolProbity score	1.97	1.87

cLIMK1i was soaked in LIMK1-KD^D460N^ apo crystals to gain structural insight into its binding mode with LIMK1 and a high-resolution X-ray structure was obtained. In the cLIMK1i-bound LIMK1 structure, cLIMK1i binds within the type I site (adenine pocket), effectively mimicking ATP interactions and stabilizing an active-like conformation of LIMK1 (Fig. 3, *B* and *C*, *D* and PDB 9Z6D, and Table 1). The 2-aminopyrimidine moiety of cLIMK1i establishes hydrogen bond interactions with the LIMK1 hinge region, specifically with residues E414, I416, and G419, displacing two water molecules near E414, I416 and K417 in the apo structure, while also interacting extensively with the surrounding hydrophobic pocket.

**Figure 4:**
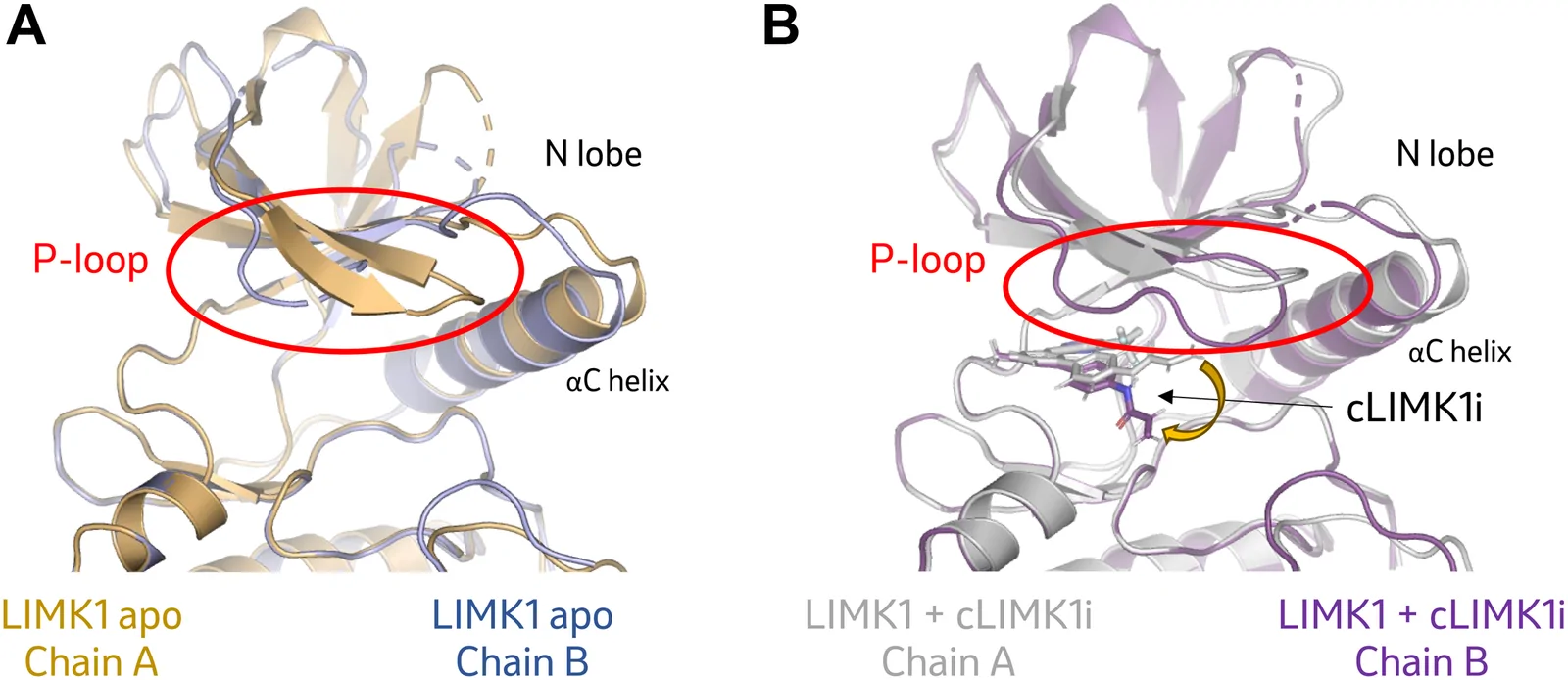
**P-loop variability between apo and cLIMK1i-bound structures.***A*, superposition of the two apo LIMK1 chains within the asymmetric unit (a.s.u.), with the P-loop highlighted by a *red oval*. P loops of chains A and B show substantial differences. *B*, superposition of the two cLIMK1i-bound LIMK1 chains in the a.s.u., showing the P-loop highlighted by a *red oval*. A *yellow arrow* indicates the acrylamide shift observed between the two chains. Chains A and B show substantial differences in the positions of both the P loop and cLIMK1i. LIMK1, LIM domain kinase 1.

In this crystal structure, cLIMK1i does not form a covalent bond with C349, despite the acrylamide moiety being positioned near the P-loop where C349 is located. Attempts to reposition the acrylamide closer to C349 by rotating cLIMK1i, or to refine the structure in the absence of cLIMK1i, failed to produce a covalent interaction. This indicates that the observed conformation—where the acrylamide is not within binding distance to C349—accurately reflects the crystal structure. The asymmetric unit contains two LIMK1–cLIMK1i complexes, Chains A and B, and neither acrylamide group is close enough to interact with C349.In Chain A, clear *2mFo-DFc* electron density defines the precise orientation of cLIMK1i relative to the P-loop. However, this orientation is inconsistent with both the 2*mFo-DFc* and *mFo-DFc* densities observed for Chain B, where the acrylamide of cLIMK1i is oriented in the opposite direction of the P-loop (Figs. 4, *A* and *B*, S6). This discrepancy between the two chains strongly suggests conformational flexibility of the LIMK1 P-loop, which in turn allows for flexibility of the cLIMK1i right hand side, as observed in the crystal structure.

The observation that LIMK1 possesses a flexible P-loop is further supported by the apo structure, which also contains two chains (A and B). In the apo form, the P-loop demonstrates similar flexibility (Fig. 4, *A* and *B*): it is better-resolved but not fully resolved, and modeled in Chain A, whereas in Chain B it is not modeled due to an electron density break between residues G346 and Q352, which renders the P-loop invisible at a *2mFo-DFc* sigma contour level of 1.0 to 2.0.

Although there are examples in the literature of covalent inhibitors not forming covalent bonds in crystal structures (29), we sought to better understand why cLIMK1i did not show binding to C349 in the crystal structure by analyzing the structure and examining modeling approaches. Based on the crystal packing, C349 is in proximity and may partially contribute to crystal contacts with symmetry-related molecules forming the crystal lattice. This interaction may influence the conformation of the P-loop, potentially alter its dynamics and lead to discrepancies with the ability of C349 to form covalent interactions with cLIMK1i in solution (Fig. S7). The conformational restraints imposed by these crystal contacts could contribute to variations in the observed flexibility and reactivity of C349, which may not fully reflect its behavior in solution or in the absence of such crystal interactions. Here, it is important to note that co-crystallization experiments of LIMK1-KD^D460N^ with cLIMK1i did not generate any crystal hits. As a result, it was not possible to determine a structure and assess whether *in-solution* pre-incubation of the inhibitor prior to crystallization would permit the structural rearrangement needed for the P-loop to move into close proximity with the acrylamide warhead and form a covalent interaction.

To investigate whether it may be possible for cLIMK1i to covalently bind to the C349 residue on the P-loop, we took a computational modeling-driven approach. Initially we ensured that standard docking could replicate the crystallographic binding pose of cLIMK1i. Using the protein preparation and Glide XP docking of the Schrödinger suite, focusing only on chain A of the structure, we were able to reproduce the crystallographic pose very reliably. We then attempted covalent docking, using CovDock from Schrödinger, but we were not able to reproduce a pose wherein the ligand was simultaneously docked to the binding site and reproducing the interactions with the Hinge region identified from the crystal structure, while being covalently bound to the C349 in the orientation in which cLIMK1i was identified in the crystal structure. To determine whether different conformations of the P-loop may be possible in the absence of the crystal contacts identified between symmetry mates for C349, we used the loop builder function of MOE. We built 200 loops, using both the PDB scanning and *de novo* building options. We found that some high scoring *de novo* loops presented the sidechain of C349 at a distance and in an orientation that could hypothetically facilitate the Michael addition to the double bond of cLIMK1i (Fig. S8, *A* and *B*). We used covalent docking again with one high scoring loop orientation and were very reliably able to reproduce the crystallographic binding pose of cLIMK1i while simultaneously covalently binding to the C349 sidechain (Fig. S9*C*).

### Biophysical characterization of cLIMK1i

**Figure 5:**
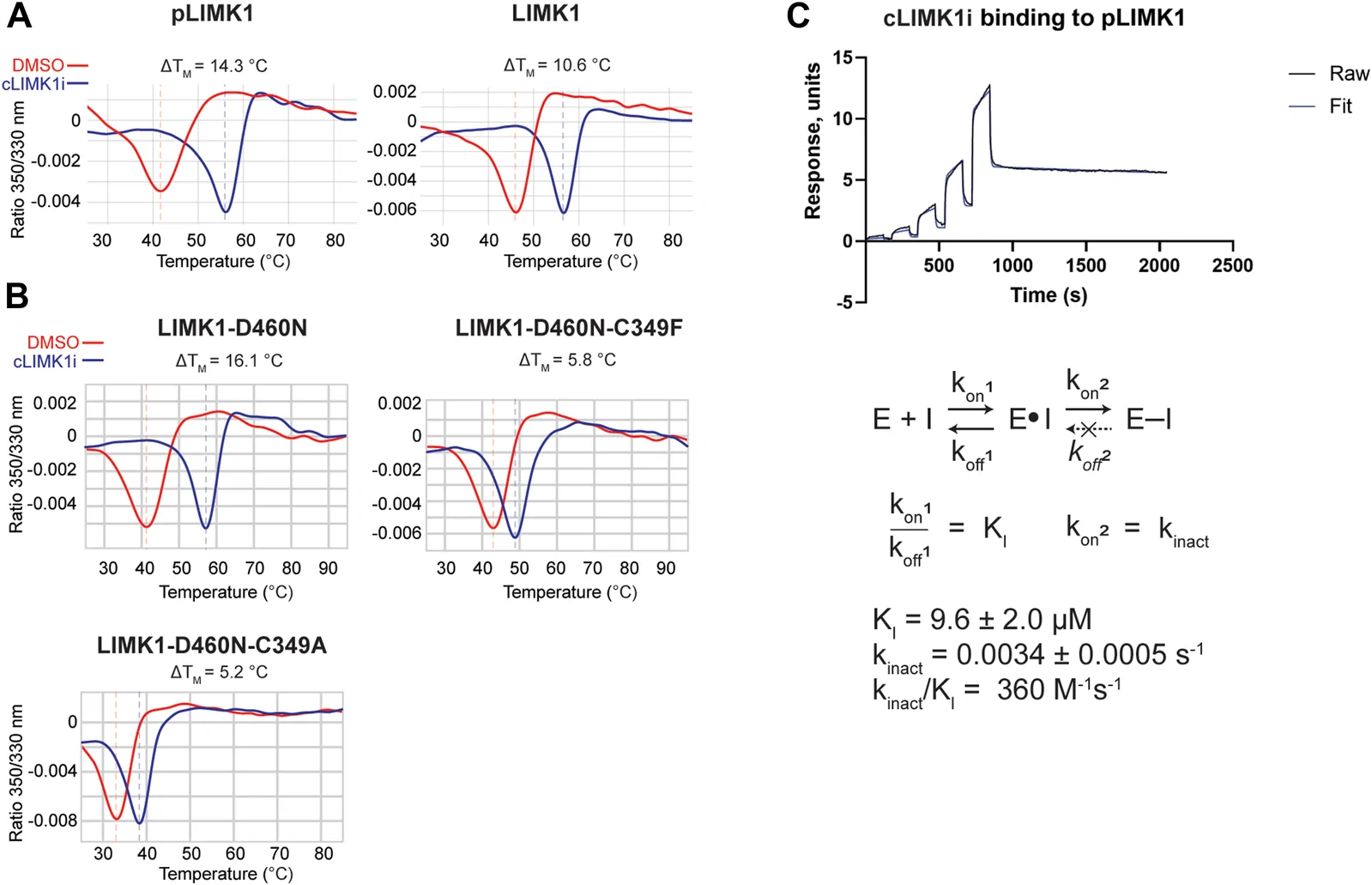
**Biophysical characterization of cLIMK1i and LIMK1 interaction.***A*, NanoDSF melting curves of LIMK1 and pLIMK1. cLIMK1i stabilizes both LIMK1 and pLIMK1 as measured by thermal stabilization on NanoDSF. Thermal melts are averages from n = 2. *B*, NanoDSF melting curves of LIMK1-D460N (n = 2), and C349F (n = 2) and C349A (n = 3) preincubated with either cLIMK1i or DMSO. cLIMK1i thermally stabilizes LIMK1 substantially more when there is a cysteine present at position 349 compared to an alanine or phenylalanine. *C*, SPR sensogram of cLIMK1i binding to pLIMK1. SPR binding data is fit to a two-step kinetic model to describe two-step covalent binding. Data is shown from one replicate, but experiment was performed as n = 2. Sensograms for both replicates can be found in Figure S10. Error in K_I_ and k_inact_ are from n = 2 data. cLIMK1i, covalent inhibitor of LIMK1; LIMK1, LIM domain kinase 1; NanoDSF, nano-differential scanning fluorimetry; SPR, surface plasmon resonance.

To validate the crystallographic assignment of cLIMK1i as a type I binder and to define the role of residue C349 in ligand engagement, the ability for cLIMK1i to stabilize both phosphorylated and unphosphorylated LIMK1 was characterized using nano-differential scanning fluorimetry (NanoDSF) (30). cLIMK1i stabilized the melting temperature of both LIMK1-KD and pLIMK1-KD by 10.6 °C and 14.3 °C, respectively, further confirming its ability to bind both phosphorylated and unphosphorylated LIMK1 (Fig. 5*A*). To determine the contribution to binding C349, two mutants of LIMK1 were generated from LIMK1-KD^D460N^: LIMK1-KD^D460N,C349A^ and LIMK1-KD^D460N,C349F^. LIMK2 contains a phenylalanine in the position equivalent to LIMK1 C349. Analytical size-exclusion chromatography showed that all constructs, including the D460N parent and the two C349 mutants, were monomeric in solution (Fig. S10), ruling out differences in oligomeric state as a confounding factor in the binding analyses. LIMK1-KD^D460N^ was stabilized by cLIMK1i with a thermal shift of 16.1 °C. The C349F and C349A mutants resulted in stabilization by cLIMK1i by only 5.8 °C and 5.2 °C, respectively (Fig. 5*B*). These results indicate that the majority of stabilization by cLIMK1i is afforded by covalent binding to C349, and not by non-covalent interactions in the type I site (Fig. 5*B*). Replacement of the cysteine by phenylalanine is not expected to affect noncovalent binding of cLIMK1i to the type I site, which is supported by the crystal structure and modeling of cLIMK1i bound to LIMK1, where the P-loop has ample flexibility to accommodate cLIMK1i and the larger size of the phenylalanine. Therefore, we attribute the change in thermal stability from cLIMK1i binding to be entirely due to the loss of covalent binding to C349.

The non-covalent binding contribution between cLIMK1i and LIMK1 was further investigated by two complementary strategies: inhibition potency using a noncovalent analog, and by SPR binding kinetics. A noncovalent analog of cLIMK1i lacking the covalent handle was tested for inhibition potency in the LIMK1 biochemical assay, and the noncovalent analog was 178-fold less potent than cLIMK1i against LIMK1 (Fig. S11). Therefore, we conclude that a substantial portion of the inhibition potency of cLIMK1i is afforded from its ability to form a covalent bond with LIMK1. Analysis of binding by SPR was employed to give further insight into the covalent and non-covalent rates of inhibition by being able to measure rate of inactivation (k_inact)_ and K_I_ of cLIMK1i. Biotinylated-pLIMK1-KD was immobilized on a neutravidin surface. Measurement of binding of cLIMK1i to biotinylated-pLIMK1-KD was performed in duplicate and was best fit with a two-step model, due to the fast formation of an enzyme-inhibitor complex (E**·**I) followed by a slower irreversible covalent complex (E‒I) (Figs. 5*C*, S11) (31, 32). The K_I_ measured by SPR demonstrates relatively fast on- and fast off-rates (k_on_1 and k_off_1), which describe a K_I_ of 9.6 ± 2.0 μm (Fig. 5*C*). The k_on_2, or k_inact_, which describes the irreversible bond forming event between cLIMK1i and pLIMK1, was measured as 0.0034 ± 0.0005 s^−1^. The off-rate k_off_2 is assumed to be 0, given the combination of biochemical and biophysical evidence which supports irreversible binding of cLIMK1i with (p)LIMK1. From SPR, the k_inact_/K_I_ was measured to be 360 M^−1^s^−1^. This modest k_inact_/K_I_ is largely driven by the low μM K_I_ for cLIMK1i to LIMK1. The SPR-derived k_inact_/K_I_ indicates that the noncovalent binding of cLIMK1i to LIMK1 is relatively weak (single-digit μm binding), while the relatively fast irreversible binding to C349 contributes greatly to its inhibitory activity and likely drives the high selectivity for inhibiting LIMK1 over LIMK2.

## Discussion

Leveraging kinome counter-screening data for historic internal kinase programs, cLIMK1i was identified as a potent and selective LIMK1 inhibitor. cLIMK1i demonstrated 206-fold selectivity for LIMK1 over LIMK2 in the biochemical assays and >11-fold selectivity in the cellular assays, which warranted further characterization into the basis of its selectivity for LIMK1. The acrylamide of cLIMK1i serves as a Michael acceptor which reacts with C349 on the P-loop of LIMK1, where no cysteine is found in LIMK2. The binding of cLIMK1i was determined to be irreversible, with NanoDSF and SPR binding studies confirming that the covalent binding of LIMK1 to C349 contributes substantially to the potency and selectivity of cLIMK1i for LIMK1 over LIMK2. Indeed, as confirmed by crystal structure, cLIMK1i binds in the type I site with its acrylamide pointing toward C349.

During the preparation of this manuscript, the discovery of a covalent LIMK1 inhibitor with selectivity over LIMK2 was described (17). This compound, SM311, was designed by introducing a covalent warhead to a dual LIMK1/LIMK2 inhibitor structurally related to BMS-5 (17, 24). Similarly to cLIMK1i described herein, SM311 showed modest noncovalent binding to LIMK1 and strong preference for inhibition of LIMK1 over LIMK2. Both SM311 and cLIMK1i inhibited a small number of kinases in an off-target kinase panel when tested at 1 μm. SM311 and cLIMK1i demonstrate comparable binding kinetics, where the k_inact_/K_I_ for SM311 was measured as approximately 20-fold higher than that of cLIMK1i. Most of this difference is attributable to each inhibitor’s K_I_. Optimizing the balance between K_I_ and k_inact_ is therefore critical for improving covalent LIMK1 inhibitors; stronger noncovalent binding with LIMK1, and subsequently covalent binding to C349 can help avoid off-target modification of other kinases, especially those that bear reactive cysteines. Crucially, we confirmed the binding mode of cLIMK1i by X-ray crystallography and modeled the covalent bond formation with C349, directly supporting the structural basis for its LIMK1 selectivity. Additionally, cLIMK1i is structurally differentiated from SM311, as well as from the BMS-5 core structure, providing an alternative avenue for covalent drug discovery efforts against LIMK1.

Collectively, these findings provide critical structural insights into LIMK1’s active-like conformation and its mode of inhibitor engagement, particularly within the ATP-binding pocket. The study highlights the intrinsic conformational flexibility of the P-loop, particularly around C349, and demonstrate how this flexibility influences covalent inhibitor binding (33, 34). The absence of covalent bond formation in the crystal structure, despite the proximity of the acrylamide moiety, underscores crystallography constraints imposed by crystal packing interactions on P-loop conformation and reactivity, emphasizing the importance of considering protein dynamics beyond static crystallographic snapshots. Furthermore, the successful development of a novel crystallization system using the LIMK1-KD^D460N^ mutant not only enabled the first high-resolution apo LIMK1 structure but also proved amenable to inhibitor soaking, establishing a robust and versatile platform for future structural studies of LIMK1 and its inhibitors. Complemented by computational modeling that revealed alternative P-loop conformations compatible with covalent binding, this integrated structural and *in silico* approach lays a strong foundation for the rational design and optimization of covalent LIMK1 inhibitors. Ultimately, these advances deepen our understanding of kinase regulation through structural plasticity and provide valuable tools to guide the development of selective, mechanism-based therapeutics targeting LIMK1.

To effectively identify a safe covalent inhibitor of LIMK1, it may be important to target the reactive cysteine of LIMK1 while minimizing the impact on the broader kinome and proteome (35). Notably, the three other kinases which cLIMK1i inhibits from the counterscreen panel, CDK7, SYK, and IRAK1, all contain reactive cysteines which have been the targets of covalent inhibitors (36, 37, 38). It is likely that cLIMK1i is engaging other reactive cysteines in the proteome, and substantial characterization and development of this molecule would be needed for clinical progression. As with other treatment options which rely on covalent reaction with a single residue, mutational resistance must be considered (39). Nonetheless, this work presents an *in vitro* characterization that establishes the feasibility of a covalent approach for selective LIMK1 targeting. We propose that cLIMK1i can find utility as a reference compound for the selective inhibition of LIMK1 over LIMK2 in both *in vitro* and *in vivo* studies.

## Experimental procedures

### Material sourcing

Chemicals were purchased from commercial suppliers and used without further purification. Echo qualified LDV plates were purchased from Beckman Coulter. Proxiplates and pCofilin Alpha SureFire Ultra Multiplex Detection Kit were purchased from Revvity. PDL-coated 384-well plates were purchased from Corning. Jump-In TRex Retargeting Kit was purchased from Life Technologies. DMEM, GlutaMax, dialyzed fetal bovine serum, Hepes for cell culturing; Minimum Essential Media Non-Essential Amino Acids, penicillin/streptomycin, Blasticidin, Geneticin, and bovine serum albumin for cell culture were purchased from Gibco. Tetracycline was purchased from EMD-Millipore. DMSO, TCEP (bond breaker), DTT, and glycerol were purchased from Sigma. 1 M MgCl_2_ and neutravidin were purchased from Fisher. BSA for biochemical assays was purchased from Gemini Bio-products. ADP Glo kit and Trypsin gold were purchased from Promega. Tris pH 7.5 for biochemical assays, Hepes pH 7.5 for SPR assays, and NaCl for SPR assays were purchased from Teknova. P20, SPR chips, HBS-EP, NHS, and EDC were purchased from Cytiva.

### Synthesis, stability, and solubility of cLIMK1i and noncovalent cLIMK1i

cLIMK1i and its noncovalent analog can be prepared by methods analogous to those described for structurally related compounds in patent application WO 2014/031,438 A2 (40). cLIMK1i was found to be soluble at 40 μm at pH 7 PBS and was stable in storage at −20 °C for over a year.

### LIMK1 cascade inhibition assays

Compounds were prepared at 2 mM concentration in DMSO in ECHO-qualified LDV plate (Labcyte). Compounds were titrated using dose response curve mode in Labcyte ECHO 655 into a white proxiplate (Revvity) to a 67.5 nl final volume in all wells. Final dose response curve was a 10-point 3-fold dilution spanning 1.7 nM to 33.2 μm. Maximum effect wells contained a final concentration of 3.3 μm TH-470, and minimum effect wells contained DMSO. Assay buffer contained 40 mM Tris pH 7.5, 20 mM MgCl_2_, and 0.01% w/v BSA. Assay reagents were prepared as three mastermixes. Enzyme mastermix contained LIMK1-dFL and assay buffer. pPAK4 mastermix contained pPAK4 and assay buffer. Cofilin-1 mastermix contained cofilin-1, ATP, and assay buffer. All dispenses were performed with a Dragonfly Discovery. First, 2 μl of enzyme mastermix was added to each well. Plate was incubated for 30 min. Then, 1 μl each of pPAK4 mastermix and cofilin-1 mastermix were added to a final volume of 4 μl. Final concentration of all reagents: 5 nM LIMK1-dFL, 5 nM pPAK4, 125 μm ATP, 5 μm cofilin-1. Reaction was incubated for 5 h. Then, 4 μl of ADP Glo reagent was added. Reaction was incubated for 1 h. Then 8 μl of kinase detection reaction was added. Reaction was incubated for 1 h. Plate was measured on Pherastar FSX (BMG Labtech) plate reader in Lum Plus mode.

### LIMK2 cascade inhibition assays

Compounds were prepared at 2 mM concentration in DMSO in ECHO-qualified LDV plate (Labcyte). Compounds were titrated using dose response curve mode in Labcyte ECHO 655 into a white proxiplate (Revvity) to a 67.5 nl final volume in all wells. Final dose response curve was a 10-point 3-fold dilution spanning 1.7 nM to 33.2 μm. Maximum effect wells contained a final concentration of 3.3 μm TH-470, and minimum effect wells contained DMSO. Assay buffer contained 40 mM Tris pH 7.5, 20 mM MgCl_2_, and 0.01% w/v BSA. Assay reagents were prepared as three mastermixes. Enzyme mastermix contained LIMK2-dFL and assay buffer. pPAK4 mastermix contained pPAK4 and assay buffer. Cofilin-1 mastermix contained cofilin-1, ATP, and assay buffer. All dispenses were performed with a Dragonfly discovery. First, 2 μl of enzyme mastermix were added to each well. Plate was incubated for 30 min. Then, 1 μl each of pPAK4 mastermix and cofilin-1 mastermix was added to a final volume of 4 μl. Final concentration of all reagents: 5 nM LIMK2-dFL, 5 nM pPAK4, 125 μm ATP, 5 μm cofilin-1. Reaction was incubated for 5 h. Then, 4 μl of ADP Glo reagent was added. Reaction was incubated for 1 h. Then 8 μl of kinase detection reaction was added. Reaction was incubated for 1 h. Plate was measured on Pherastar FSX plate reader in Lum Plus mode.

### HEK293T expressing LIMK1/LIMK2 inhibition of cofilin-1 phosphorylation

HEK293 cell lines overexpressing full-length human WT LIMK1 (accession number NM_002314.4) or LIMK2 (accession number NM_005569.4) upon tetracycline treatment were generated using the Jump-In TRex Retargeting Kit (Life Technologies #A15008) following the manufacturer’s protocol.

Jump-In Trex HEK293 cells were cultured in DMEM GlutaMax (Gibco #10569-010) supplemented with 10% dialyzed fetal bovine serum (dFBS, Gibco #A33820-01), 10mM Hepes (Gibco #15630-080), Minimum Essential Media Non-Essential Amino Acids (Gibco #11140-050), 1x penicillin/streptomycin (Gibco # 15140-122), 5 μg/ml Blasticidin (Gibo #A1113903), 2 mg/ml Geneticin (Gibco #10131-027), and incubated at 37 °C in a humidified atmosphere containing 5% CO_2_. To induce LIMK1 or LIMK2 overexpression, cells were seeded at 6000 cells/well into white PDL-coated 384-well plates (Corning #354661) and dosed with 1 μg/ml tetracycline (EMD-Millipore #58346) for 24 h prior to compound testing.

Test compounds were dissolved in DMSO at a stock concentration of 10 mM. For cellular assays, the 10 mM stock was pre-plated into polypropylene plates using an ECHO 650 Series liquid handler (Beckman Coulter) and then diluted with cell media containing 0.25% BSA instead of dFBS immediately prior to dosing on cells. In cell plates, media containing dFBS was replaced with media containing 0.25% BSA (Gibco, #15260-037), and cLIMK1i was dosed in a 10-point titration series in wells of cells for 3 h at 37 °C, 5% CO_2_. Additional wells of cells were dosed with either 0.1% DMSO or 10 μm TH-470 to serve as Minimum-Effect or Maximum-Effect controls, respectively. After compound incubation, cell media and test compounds were removed by gentle plate centrifugation.

Cells were lysed and the level of phospho-Cofilin (Ser3) and total cofilin in cell lysates was measured using the Alpha SureFire Ultra Multiplex Detection Kit (Revvity #MPSU-PTCOF) following the manufacturer’s protocol. AlphaLISA signal from lysates was detected using a Pherastar FSX platereader (BMG LabTech) equipped with an AlphaPlex filter module (545 and 615 nm emissions for Total and Phospho-cofilin signal, respectively) in simultaneous detection mode. The ratio of phospho-Cofilin signal to total Cofilin signal was calculated for each well and normalized as percent effect relative to the DMSO and TH-470 control wells. The 10-point titration series of cLIMK1i was fit with a 4-parameter curve to determine compound IC_50_.

### Inhibition time course experiment with pLIMK1-KD

Two mastermixes were prepared: enzyme mastermix contains pLIMK1-KD and assay buffer (40 mM Tris pH 7.5, 20 mM MgCl_2_, and 0.01% w/v BSA). Reagent mastermix contains ATP and cofilin-1. Reactions were set up with varying concentrations of cLIMK1i. cLIMK1i was added to each reaction vessel starting from a 10 mM stock in DMSO to final concentrations of 1000, 500, 250, 125, 62.5, and 31.25 nM. DMSO concentrations were not matched across samples, due to the 1000 nM cLIMK1i sample containing only 0.01% DMSO. The enzyme and reagent mastermixes were added in succession to each sample with varying cLIMK1i concentrations, to initiate the reaction. Final concentrations were 5 nM pLIMK1-KD, 1 μm cofilin-1, and 50 μm ATP. The total reaction volume was 10 μl. The timepoints were run by quenching all timepoints at the same time with ADP Glo reagent, so the 20-min timepoint was started first, then 10 min later, the 10-min timepoint was started, then every 2 min, another timepoint was started. At 20 min, all samples were quenched with 10 μl ADP Glo reagent, and following a 1-h incubation, 20 μl of kinase detection reagent was added.16 μl of sample was transferred to a white proxiplate, and wells were measured on Pherastar FSX plate reader in Lum Plus mode.

### Jump dilution experiment

500 nM pLIMK1-KD was pre-incubated for 30 min with either DMSO, 2 μm cLIMK1i, or 16.6 μm BMS-5 in assay buffer (total volume = 30 uL, 3% DMSO). This mixture was diluted 500x to a final concentration of 1 nM pLIMK1-KD, by addition of cofilin-1 (final concentration = 1 μm), ATP, and assay buffer (40 mM Tris pH 7.5, 20 mM MgCl_2_, and 0.01% w/v BSA). The total reaction volume was 10 μl. The timepoints were run by quenching all timepoints at the same time with ADP Glo reagent, so the 210 min timepoint was started first, then 150 min later, the 60 min timepoint was started, then 20 min later, the 40 min timepoint was started, *etc*. 210 min after the first timepoint was started, all samples were quenched with 10 μl ADP Glo reagent, and following a 1 h incubation, 20 μl of kinase detection reagent was added.16 μl of sample was transferred to a white proxiplate, and wells were measured on Pherastar FSX plate reader in Lum Plus mode.

### Intact MS of LIMK1-KD treated with cLIMK1i

A mixture containing 5 μm LIMK1-KD was incubated with either DMSO or 10 μm cLIMK1i in assay buffer (40 mM Tris pH 7.5, 20 mM MgCl_2_), with a final volume of 30 μl and a final concentration of DMSO of 0.1%. The mixture was incubated for 30 min prior to analysis by intact protein LC-MS. 20 μl of sample was injected on LC-MS. Buffer A: water + 0.1% trifluoroacetic acid. Buffer B: acetonitrile + 0.1% TFA. Samples were separated on LC using a Vydac 214TP 5 μm C4 column (50 × 2.1 mm). The gradient was run at 0.3 ml/min 0 min: 2% A, 1 min: 2% A, 9 min: 90% A, 10.5 min: 90% A, 10.6 min: 2% A, 14.1 min 2% A. The mass spectrometer used was a Thermo Fisher Scientific Q Exactive HF. Mass spectrometer conditions: full MS scan mode, positive ion mode, in-course CID: 20 eV, resolution: 15,000, AGC target: 3e6, Maximum IT: 200 ms, Scan range: 700 to 4000 m/z.

To analyze data, the LIMK1 elution was identified by examining UV chromatogram. All mass scans from the corresponding peak in LC-MS chromatogram were selected and applied to Promass software, using default settings aside from the following: baseline removal: 0.2, S/N: 1.5.

### Trypsin digest of LIMK1-KD treated with cLIMK1i

A mixture containing 5 μm LIMK1-KD was incubated with either DMSO or 10 μm cLIMK1i in assay buffer (40 mM Tris pH 7.5, 20 mM MgCl_2_), with a final volume of 25 μl and a final concentration of DMSO of 0.1%. The mixture was incubated for 45 min. Then, 2.5 uL of quench buffer was added 2.5 uL of the LIMK1-KD mixture to a final concentration of 3 M guanidine HCl, 50 mM Tris pH 8, and 2 mM DTT. Sample was incubated at 37 °C for 45 min. Then 20 uL of dilution buffer (50 mM Tris pH 8, 1 mM CaCl_2_) and sequence grade trypsin (Promega) was added to a final concentration of 0.02 μm. Digest proceeded for 16 h at ambient temperature. Digest was halted by addition of formic acid to reach pH 3, and samples were kept frozen at −80 °C until ready for analysis by LC-MS. 20 μl of each sample was injected on LC-MS. Buffer A: water + 0.1% trifluoroacetic acid. Buffer B: acetonitrile + 0.1% TFA. Samples were separated on LC using a Vydac 214TP 5 μm C4 column (50 × 2.1 mm). The gradient was run at 0.3 ml/min 0 min: 2% A, 1 min: 2% A, 9 min: 90% A, 10.5 min: 90% A, 10.6 min: 2% A, 14.1 min 2% A. The mass spectrometer used was a Thermo Fisher Scientific Q Exactive HF. Mass spectrometer conditions: full MS to ddMS^2^ scan mode, positive ion mode, full MS scan range: 200 to 2000 m/z, AGC target: 2e5, resolution: 120,000, maximum IT: 100 ms. dd-MS2 conditions: resolution: 30,000, AGC target: 1e5, maximum IT: 50 ms, loop count: 5, TopN: 5, isolation window: 4.0 m/z, stepped NCE: 30, 35, 40. dd settings: minimum AGC: 1e3, intensity threshold: 3e4, peptide match: preferred, exclude isotope: on, dynamic exclusion: 10s.

### cLIMK1i binding to pLIMK1-KD on biacore

Cytiva Series S CM5 sensor chip was docked into the Biacore 8K+, and the pLIMK1 surface was prepared according to the following protocol. Running buffer was 10 mM Hepes pH 7.4, 150 mM NaCl, 0.05% w/v P20, 1 mM TCEP, and 2% DMSO. Neutravidin was immobilized to the chip surface using NHS/EDC coupling. The flow rate used was 10 μl/min, EDC/NHS mixture was injected for 300 s, then neutravidin (20 μg/ml in sodium acetate (10 mM, pH 4.5) was injected for 300 s, followed by neutralization by ethanolamine (1 M, pH 8.5). Then, biotinylated-pLIMK1 (20 μg/ml in HBS-EP) was flowed across the surface for 100 s at 10 μl/min. Compounds were injected in single-cycle kinetic mode at 50 μl/min for 120 s, followed by a 120 s dissociation period, and following the top concentration injection, a 1200 s dissociation period. Concentrations of compound injected were 123, 370, 1111, 3333, and 10,000 nM. Data analysis was performed using Cytiva Insight software, using the two-state model.

### pLIMK1-KD reversibility by Biacore

Cytiva Series S CM5 sensor chip was docked into the Biacore 8K+, and the pLIMK1 surface was prepared according to the following protocol. Running buffer was 10 mM Hepes pH 7.4, 150 mM NaCl, 0.05% w/v P20, 1 mM TCEP, and 2% DMSO. Neutravidin is immobilized to chip surface using NHS/EDC coupling. The flow rate used is 10 μl/min, EDC/NHS mixture is injected for 300 s, then neutravidin (20 μg/ml in sodium acetate (10 mM, pH 4.5) is injected for 300 s, followed by neutralization by ethanolamine (1 M, pH 8.5). Then, biotinylated-pLIMK1 (20 μg/ml in HBS-EP) is flowed across surface for 100 s at 10 μl/min.

To establish baseline binding of BMS-5 (including a blank correction) to each spot, 1 μm BMS-5 was flowed for 90 s at 30 μl/min. Then, two spots were treated with 10 μm cLIMK1i, flowed at 10 μl/min for 900s. Subsequently, every 2.5 h, BMS-5 (including a blank correction) was injected. 7 cycles of this were performed.

Data was analyzed by plotting the maximum response of each BMS-5 injection and converting to the surface activity based on the biotinylated-pLIMK1 that was immobilized. Then, the relative surface activity was calculated by dividing the surface occupancy at each timepoint by the initial surface activity, to normalize the signal from each surface. Each set of duplicates (surface either treated with cLIMK1i or untreated) was then averaged. The average relative surface occupancy of cLIMK1i treated surfaces was divided by the average relative surface activity of the untreated surfaces to generate the corrected surface activity of cLIMK1i treated surface. This takes into account the loss binding response that occurred without cLIMK1i treatment. This corrected surface activity is what is plotted in Figure 2*D*.

### (p)LIMK1 cLIMK1i time-course LC-MS assay

A mixture containing 5 μm LIMK1-KD was incubated with either DMSO or 10 μm cLIMK1i in assay buffer (40 mM Tris pH 7.5, 20 mM MgCl_2_), with a final volume of 150 μl and a final concentration of DMSO of 0.1%. The mixture was immediately injected on LC-MS for intact protein LC-MS. Additional samples were injected every 30 min 20 μl of sample was injected on LC-MS. Buffer A: water + 0.1% trifluoroacetic acid. Buffer B: acetonitrile + 0.1% TFA. Samples were separated on LC using a Vydac 214TP 5 μm C4 column (50 × 2.1 mm). The gradient was run at 0.3 ml/min 0 min: 2% A, 1 min: 2% A, 9 min: 90% A, 10.5 min: 90% A, 10.6 min: 2% A, 14.1 min 2% A. The mass spectrometer used was a Thermo Fisher Scientific Q Exactive HF. Mass spectrometer conditions: full MS scan mode, positive ion mode, in-course CID: 20 eV, resolution: 15,000, AGC target: 3e6, Maximum IT: 200 ms, Scan range: 700 to 4000 m/z.

To analyze the data, LIMK1 elution was identified by examining UV chromatogram. All mass scans from the corresponding peak in LC-MS chromatogram were selected and applied to Promass software, using default settings aside from the following: baseline removal: 0.2, S/N: 1.5.

### NanoDSF of WT (p)LIMK1, LIMK1-KD^D460N^, LIMK1-KD^D460N,C349F^, and LIMK1-KD^D460N,C349A^

Each LIMK1 protein construct at 2.5 μm was incubated in buffer (25 mM Hepes pH 7.5, 150 mM NaCl, 1 mM TCEP, 5% glycerol) with compounds at 25 μm. Compounds were prepared from 10 mM stocks in DMSO and were diluted into the mixture at a final DMSO concentration of 1%. Compounds were incubated with LIMK1-KD for 30 min before running the temperature gradient (1.5 °C/min, from 25-85 or 95 °C). All experiments were performed as n = 2.

### Protein expression and purification

LIMK1 catalytic domain DNA comprised of residues 329 to 638 with an N-terminal hexahistidine (His_6_) tag, a tobacco etch virus (TEV) cleavage site and a D460N kinase-inactivating point mutation was cloned into pBAC1 vector (LIMK1-KD^D460N^). Additional LIMK1 catalytic domain mutants (C349F and C349A) were similarly designed. WT LIMK2 kinase domain, consisting of residues 330 to 632 with N-terminal cleavable His_6_-TEV was also cloned in pBAC1. Proteins were expressed at 27 °C using the baculovirus expression vector system in *Spodoptera frugiperda* (Sf21) insect cells (obtained commercially from Kemp Biotechnologies, Inc). Cells were infected at a density of 2.0 × 10^6^ cells/ml at an estimated MOI of 2.0 pfu from Baculovirus Infected Insect Cells. After 72 h, cultures were centrifuged at 3400*g* for 15 min at 4 °C in a Beckman JS5.0 rotor and stored at −80 °C.

Cell pellet from 5L culture was thawed and resuspended in 300 ml Lysis Buffer consisting of 25 mM Hepes pH 7.5, 0.5 M NaCl, 20 mM imidazole, 1 mM TCEP, Roche EDTA-free Complete protease inhibitor tablets (1 tablet/50 ml buffer), DNase (50 U/ml) and manually homogenized. Cell suspension was lysed by sonication on ice using Qsonica Q700, 0.5 inch tip, amplitude 25 (∼42 W) for two cycles of 1.5 min each and 15 min break between cycles. The lysate was then ultracentrifuged at 36,000 rpm (100,000*g*) for 45 min, 4 °C in a Ti40 rotor to sediment loose cell debris.

All subsequent purification steps were performed at 4 °C. Immobilized metal affinity chromatography (IMAC) was performed using Ni-NTA 5 ml HisTrap FF Crude column (Cytiva). The cleared lysate was applied to 5 ml resin and washed with 20 column volumes Wash Buffer of 25 mM Hepes pH 7.5, 0.5 M NaCl, 20 mM imidazole, 5% glycerol, 1 mM TCEP. Protein was eluted with a 10-column volume gradient from 0 to 100% Elution Buffer of 25 mM Hepes pH 7.5, 0.5 M NaCl, 500 mM imidazole, 5% glycerol, 1 mM TCEP. Fractions containing LIMK proteins were pooled, and TEV was added at a mass ratio of 10:1 w/w of protein to protease. Cleavage of N-terminal His tag was performed overnight at 4 °C during dialysis against IMAC Wash Buffer. Contaminating proteins, cleaved tag, and TEV protease were removed with a subtractive IMAC step. Cleaved LIMK1 protein was recovered in the flow-through when loading into a 5 ml HisTrap FF Crude column. Protein devoid of tag was concentrated using centrifugal filter with MWCO 10 kDa (Amicon Ultra, Millipore Sigma) and injected to a HiLoad 26/60 Superdex 200 pg column (Cytiva) equilibrated with SEC Buffer 25 mM Hepes pH 7.5, 0.5 M NaCl, 5% glycerol, 1 mM TCEP. Final yields of approximately 2.8 mg/L LIMK1-KD variants and 0.5 mg/L LIMK2 kinase domain were recovered, respectively, and LIMK1-KD^D460N^ was further concentrated for crystallization experiments.

Full-length human cofilin (1–166) was expressed with an N-terminal His_6_ tag and TEV cleavage site in BL21(DE3) *Escherichia. coli*. Cells were cultured in LB media in shake flasks at 37 °C and harvested 18 h post-induction. Cell pellets were resuspended in Buffer A (50 mM Tris, pH 7.2; 150 mM NaCl; 1 mM DTT; 10 mM L-Methionine) and lysed with two passes through an emulsifier prior to centrifugation at 35,000*g* for 1 h to remove debris. Supernatant was loaded onto HisTrap crude FF resin (Cytiva), washed with Buffer A supplemented with 20 mM imidazole, and bound protein eluted with Buffer A containing 300 mM imidazole. Elution fractions were dialyzed against Buffer A in the presence of TEV protease overnight and liberated N-terminal peptide and TEV protease were removed by subtractive IMAC using resin and buffers described above. Finally, monomeric protein was separated on a S75 26/600 gel filtration column (Cytiva) equilibrated in Buffer A.

To phosphorylate LIMK1-KD, purified human LIMK1-KD was incubated with human PAK4 KD (300–591) at a 4:1 mass ratio in Buffer B including 2 mM ATP and 5 mM MgCl_2_. After a 5-h incubation, His-tagged LIMK1-KD was separated from untagged PAK4 *via* second capture on HisTrap Crude column and pLIMK1-KD was further polished by a final gel filtration step.

Biotinylated LIMK1-KD was expressed as a His_6_-GST-TEV-AVI-LIMK1 fusion protein in pBAC1 vector in Sf21 cells cultured in Sf900 II media. Cells were harvested by centrifugation 72 h post infection, lysed by emulsification after resuspension in Buffer B, and loaded onto HisTrap FF column. Bound protein was washed with Buffer B supplemented with 50 mM imidazole and eluted during 20 column volume gradient to 500 mM imidazole. Protein was biotinylated with BirA enzyme after addition of 10 mM ATP, 10 mM magnesium acetate, and 50 uM biotin. Solution incubated for 2 h and GST tag was cleaved by TEV protease during overnight dialysis against Buffer B. GST and his-tagged BirA and TEV were removed from LIMK1 *via* subtractive IMAC on a HisTrap FF column. Finally, protein was polished on a Superdex 200 26/60 column equilibrated in Buffer B. pLIMK1 was prepared as described above, but with His-tagged PAK4 which was removed by subtractive IMAC prior to gel filtration.

The gene for full-length human LIMK2 contained an N-terminal His_6_ sequence followed by GST tag, TEV cleavage site, and finally LIMK2 (1–638) in pFastBac1 vector. Sf21 cells grown in Sf-900 II medium with 10% fetal calf serum were harvested 64 h post-infection. Cell pellet was resuspended in Buffer C (50 mM Tris, pH 7.5; 500 mM NaCl; 20% glycerol, 1 mM TCEP; 0.5 mM EDTA; 0.1% CHAPS) and lysed by disperser. Lysate incubated with Glutathione Sepharose 4 FF (Cytiva) and beads were subsequently washed with Buffer C before elution with Buffer C supplemented with 10 mM glutathione. The N-terminal tag was liberated by TEV protease during overnight dialysis against Buffer C and separated from LIMK2 by subtractive GST affinity chromatography. Monomeric protein was isolated by gel filtration on a Superdex 200 16/60 column equilibrated in Buffer C.

### Structure determination

For crystallization, the apo LIMK1 KD (residues 329–638) carrying the D460N point mutation (LIMK1-KD^D460N^) was screened at 7.4 mg/ml using commercial high-throughput crystallization screens. Rod-shaped crystals grew in Morpheus (Molecular Dimensions) HT-96 conditions A8 and D12. Condition A8 consisted of 0.06 M divalents, 0.1 M Buffer System 2, pH 7.5, and 37.5% (v/v) Precipitant Mix 4. Condition D12 consisted of 0.12 M alcohols, 0.1 M Buffer System 3, pH 8.5, and 37.5% Precipitant Mix 4.

Hanging drops were set up with 1 μl protein solution mixed with 1 μl precipitant over a 500 μl reservoir and incubated at 20 °C. Crystals reached full size within 3 to 4 days; however, significant ‘skin’ formation was observed at the top of the drop, most likely due to precipitated protein. This could not be optimized, and the crystals often grew attached to the ‘skin’. As a result, they had to be carefully removed by cutting through the ‘skin’ before cryoprotecting and freezing. For complex formation, apo LIMK1-KD^D460N^ crystals were soaked in 0.5 mM cLIMK1i for 3 days at 20 °C after reaching maximum size, and then harvested. All crystals were cryoprotected with 20% (v/v) ethylene glycol prior to harvesting.

X-ray diffraction data were acquired at the Canadian Light Source , beamline CMCF-BM, on a PILATUS3 6M detector. Full datasets were collected from single crystals of both LIMK1-KD^D460N^ and LIMK1-KD^D460N^ + c LIMK1i. Raw diffraction images were processed using the autoPROC pipeline (Global Phasing) (41). Data processing and analysis with the autoPROC toolbox (42). Both structures were determined by molecular replacement using Phaser (43) refined using Phenix Refine (44, 45) and built using Coot (46). Structure figures were prepared using Pymol (The PyMOL Molecular Graphics System, Version 3.0 Schrödinger, LLC.).

### Molecular modeling to support covalent binding of cLIMK1i; loop modeling and docking

Schrodinger Maestro version 14.0.134 was used to prepare the LIMK1-KD^D460N^ + cLIMK1i crystal structure for docking (47). Standard Glide XP docking was used to recapitulate the non-covalent cLIMK1i docking mode observed in the crystal structure (48). CovDock was used to perform covalent docking using Michael addition (49), but it could not replicate the binding pose from the crystal structure. Subsequently, MOE 2024.06 (50) was used to prepare the structure and, after removing the ligand, the Loop Builder protocol was used to rebuild the loop from position 347 to 351 drawing from both PDB search of loops and *de novo* build options. The top 200 loops were selected and monitored for the distance between the sulfur atoms of the Cys349 sidechain to the C of the allyl group of the cLIMK1i. There was a distribution of distances from 1 Å to 11.5 Å. A loop was selected that showed a distance of ∼4.4 Å between the sulfur atom of the cysteine sidechain and the ligand. This structure was transferred to Schrodinger Maestro and prepared as described above. The CovDock experiment was run, and the binding pose of the cLIMK1i ligand was replicated faithfully (Fig. S7).

## Data availability

All data associated with this study are available in the article and supporting information. The final coordinates of the crystal structures are deposited in the PDB with accession codes 9Z6D and 9Z6E as of the date of publication. The paper does not report original code.

## Supporting information

This article contains supporting information.

## Conflict of interest

The authors declare that they have no conflicts of interest with the contents of this article. All authors edited and approved the final version of the manuscript.
