# Supplementary material for: A covalent inhibitor targeting Cys-349 of LIMK1 confers selectivity over LIMK2
Source: J Biol Chem. 2026 Jul 13;302(9):113322. doi: 10.1016/j.jbc.2026.113322 (PMC13519618; doi:10.1016/j.jbc.2026.113322)
Supplement: Supplementary file 1 [file mmc1.docx]

Supplementary materials for:

**A covalent inhibitor targeting Cys-349 of LIMK1 confers selectivity over LIMK2**

Jon B Patteson*, Ioannis Manolaridis*, Mee Ra Hong, Jennifer M Johnston, Samaneh Mesbahi-Vasey, Michael C Gregory, Daniel V Iwamoto, John C Reid, John M Sanders, Ditte Lovatt, Terrence P McDonald, Valerie W Shurtleff, Sandra B. Gabelli and Marina Bukhtiyarova

*These authors contributed equally to this work

**Table of contents**

[Supplemental Figure 1. Clustal Omega alignment of LIMK1 and LIMK2	3](#_Toc179710019)

[Supplemental Figure 2. LIMK1/2 cascade assay overview.	4](#_Toc1945864484)

[Supplemental Figure 3: cLIMK1i inhibition of kinases from the broad kinase panel.	5](#_Toc1997491961)

[Supplemental Figure 4. cLIMK1i reacts with both LIMK1 and pLIMK1 at similar rates.	6](#_Toc1782299597)

[Supplemental Figure 5. Timecourse inhibition of cLIMK1i. Two independent replicates of a t	7](#_Toc1330200125)

[Supplemental Figure 6. Detail on SPR-based reversibility study.	8](#_Toc637292035)

[Supplemental Figure 7. cLIMK1i electron density for the two complex molecules in the asymmetric unit.	9](#_Toc919160777)

[Supplemental Figure 8. Crystal contacts that form the LIMK1-KDD460N crystal lattice.	10](#_Toc349709430)

[Supplemental Figure 9. Structural modeling.	11](#_Toc189295927)

[Supplemental Figure 10. Analytical size exclusion chromatography	12](#_Toc2012961050)

[Supplemental Figure 11. Inhibition potency of the noncovalent analog of cLIMK1i.	13](#_Toc1073125754)

[Supplemental Figure 12. Additional information on cLIMK1i binding	14](#_Toc615500746)

[Supplemental Figure 13. SDS-PAGE gels of all proteins used in experiments.	15](#_Toc805692134)

**Supplemental Figure 1**. Clustal Omega alignment of LIMK1 and LIMK2.

**Supplemental Figure 2**. LIMK1/2 cascade assay overview. A) Basis of LIMK1 cascade assay. Phosphorylated PAK4 (pPAK4), LIMK1, and cofilin-1 are incubated. pPAK4 phosphorylates LIMK1, which allows for phosphorylation of cofilin-1. Assay detection system is ADP Glo, which measures the concentration of ADP formed during the reaction. Due to the excess of cofilin-1 over LIMK1 (5 μM vs 5 nM), the ADP Glo readout is primarily due to phosphorylation of cofilin-1. B) Readout from time course of cascade assay containing LIMK1 and pPAK4, or just LIMK1 or just pPAK4. Luminescence signal at 4 hour timepoint provides sufficient signal-to-background and signal is almost entirely from phosphorylated LIMK1 phosphorylating cofilin-1.

**Supplemental Figure 3**: cLIMK1i inhibition of kinases from the broad kinase panel. cLIMK1i was tested at 1 μM. Values listed for each kinase are % inhibition.

**Supplemental Figure 4**. cLIMK1i reacts with both LIMK1 and pLIMK1 at similar rates. Intact mass spectrometry was used to measure cLIMK1i addition to LIMK1 and pLIMK1

**Supplemental Figure 5**. Timecourse inhibition of cLIMK1i. Two independent replicates of a time course of pLIMK1-KD phosphorylation of cofilin-1 at varying concentrations of cLIMK1i. Reaction progress is completely stopped by as little as 250 nM cLIMK1i at 20 min.

**Supplemental Figure 6**. Detail on SPR-based reversibility study. Four spots were used with pLIMK1-KD immobilized. Two spots were to be treated with cLIMK1i, and two spots were to be treated with buffer. First, all four spots were assessed with BMS-5 binding to determine the maximal response. A. Left: Sensograms of BMS-5 binding prior to treatment of pLIMK1-KD with either cLIMK1i or buffer. Note that response of BMS-5 binding is taken 5 seconds after start of dissociation segment of the sensogram. Middle: sensograms of BMS-5 binding immediately after cLIMK1i or buffer treatment. Right: Final timepoint of BMS-5 binding to conclude experiment. B. Data analysis to produce figure shown in main text figure D. Left: Percent occupancy (or level of occupancy, or surface activity) is calculated by dividing the measured binding response by the theoretical binding response based on the amount of immobilized pLIMK1-KD on each spot. Middle: The initial percent occupancy timepoint was set to 100%, and all subsequent timepoints were normalized relative to the initial timepoint. Note the loss in response over time for buffer-treated pLIMK1-KD, which indicates unavailability of proteins to bind BMS-5. Right: Therefore, we normalized the percent occupancy at each timepoint by dividing the relative percent response of cLIMK1i-treated surface to the relative percent response of buffer-treated surface (data coming from middle panel). The result of this transformation accounts for the loss of signal that occurs over time naturally at pLIMK1 remains immobilized on the surface.

**Supplemental Figure 7**. cLIMK1i electron density for the two complex molecules in the asymmetric unit. A) 2*mFo-DFc* contoured at 1.0 sigma for cLIMK1i in Chain A post refinement. B) Refinement output of Chain B with 2*mFo-DFc* contoured at 1.0 sigma and *mFo-DFc* contoured at 3.5 sigma when docking cLIMK1i in identical conformation to Chain A. C) Refinement output of Chain B with *mFo-DFc* contoured at 3.5 sigma when refining in the absence of cLIMK1i. The inhibitor (green sticks) is docked into the difference density post refinement for clarity. D) Refinement output of Chain B with 2*mFo-DFc* contoured at 1.0 sigma and *mFo-DFc* contoured at 3.5 sigma when docking cLIMK1i in identical pose to Figure 4C.

**Supplemental Figure 8**. Crystal contacts that form the LIMK1-KD^D460N^ crystal lattice. A) Crystal contact between LIMK1-KD^D460N^ Chain A and a symmetry molecule, partly mediated by a C349-Water interaction. B) Crystal contact between LIMK1 Chain B and a symmetry molecule, mediated by a C349-Water interaction.

**Supplemental Figure 9**. Structural modeling bridges biochemical and crystallographic results. **A)** Histogram of distances between the ligand and the sulfur atom of C349 observed from 200 rebuilt loops. **B)** 200 rebuilt loops for GKG**C_349_**FGQ colored in green ribbon, with C349 shown in thin sticks and the ligand shown in white sticks for reference, though it was removed for the loop building process. **C)** Figure showing covalently docked pose of cLIMK1i (cLIMK1 modeled) bound to C349 and docked into the binding site of LIMK1-KD^D460N^, reliably replicating the crystallographic binding interactions from the structure. The original crystallographic structure is shown in grey, the P-loop shown in blue and the hinge region highlighted in magenta, as in figure 3C. The modeled structure and docked cLIMK1i (cLIMK1i modeled) are shown in teal ribbon and sticks. Modelled cLIMK1i overlaps almost exactly with the crystal binding pose (white sticks), while forming a covalent bond to the P-loop C349 (sidechain shown in sticks)***.***

**Supplemental Figure 10**. Analytical size exclusion chromatography traces of proteins used for NanoDSF experiments. All constructs show identical masses, indicating any changes in binding of cLIMK1i are not due to differences in oligomeric structure.

**Supplemental Figure 11**. Inhibition potency of the noncovalent analog of cLIMK1i. A) Structures of cLIMK1i and the noncovalent analog. Noncovalent analog lacks the Michael acceptor, as well as a methyl group as noted. B) Inhibition potency curves for noncovalent analog. Noncovalent LIMK1 inhibitor was tested in duplicate. One representative inhibition potency curve is shown for cLIMK1i is shown (all replicates are shown in main text figure 1).

**Supplemental Figure 12**. Additional information on cLIMK1i binding to pLIMK1-KD by SPR. A. Second replicate of cLIMK1i binding to pLIMK1i by SPR, fit with two-step kinetics. B. One-step kinetics fit of binding data shows poor fit. The data is better fit by a two-step model.

# **Supplemental Figure 13.** SDS-PAGE gels of all proteins used in experiments.
